# Sclerostin downregulation in aseptic loosening

**DOI:** 10.1302/2046-3758.1511.BJR-2025-0796.R1

**Published:** 2026-11-03

**Authors:** Assil-Ramin Alimy, Eva Goedecke, Ana Ocokoljic, Christian Ries, Frank Timo Beil, Tim Rolvien

**Affiliations:** 1 Department of Trauma and Orthopaedic Surgery, University Medical Center Hamburg-Eppendorf, Hamburg, Germany; 2 Helios ENDO-Klinik Hamburg, Hamburg, Germany

**Keywords:** Arthroplasty, Periprosthetic, Revision, Osteocytes, Sclerostin, Aseptic loosening, Synovial fluid, primary osteoarthritis, biomarkers, Trabecular bone, revision surgeries, histomorphometry, CRP, backscattered electron imaging

## Abstract

**Aims:**

Aseptic loosening is one of the leading indications for revision surgery after total hip and knee arthroplasty, yet its diagnosis and underlying mechanisms remain poorly defined. In this exploratory study, we investigated synovial fluid biomarkers and periprosthetic bone tissue characteristics to identify molecular signatures associated with aseptic loosening.

**Methods:**

A total of 40 patients were included across three groups: primary osteoarthritis (OA; n = 13), aseptic loosening (n = 15), and other aseptic revisions (n = 12). Routine blood tests and synovial cytology were obtained. Synovial fluid underwent targeted proteomic profiling. Trabecular bone was assessed by histomorphometry, quantitative backscattered electron imaging, and immunofluorescence.

**Results:**

Routine blood (CRP, white blood cell (WBC) count) and synovial (WBC count and differential) analyses showed no differences between groups, underscoring their limited diagnostic utility. In contrast, bone-focused multiplex synovial fluid profiling identified sclerostin as the strongest discriminatory marker, with significantly lower levels in aseptic loosening than in OA and other aseptic revisions. Dickkopf-1 showed a concordant but weaker reduction, reaching significance versus OA. Histomorphometric analysis of periprosthetic trabecular bone samples demonstrated preserved microarchitecture and turnover across groups. However, quantitative backscattered electron imaging revealed enlarged osteocyte lacunae specifically in aseptic loosening. Immunofluorescence confirmed reduced sclerostin expression within periprosthetic bone, mirroring synovial fluid findings. Synovial sclerostin demonstrated promising diagnostic potential, achieving an area under the curve (AUC) of 0.82 for differentiating aseptic loosening from other revision indications.

**Conclusion:**

This study showcases osteocyte dysregulation as a key feature of aseptic loosening, and suggests that reduced osteocyte-derived sclerostin may reflect adaptive responses to abnormal mechanical and inflammatory stimuli at the implant-bone interface. Larger prospective studies are required to validate these findings, but osteocyte-derived sclerostin may represent a promising biomarker and potential therapeutic target in aseptic loosening.

Cite this article: *Bone Joint Res* 2026;15(11):1307–1316.

## Article focus

- Define molecular signatures of aseptic loosening using synovial fluid proteomics and periprosthetic bone tissue analyses.
- Compare aseptic loosening with primary osteoarthritis (OA) and other aseptic revision indications.

## Key messages

- Routine blood and synovial cytology are not useful for identifying aseptic loosening.
- Synovial sclerostin is reduced in aseptic loosening versus OA and other aseptic revision, whereas Dickkopf-1 shows a concordant but weaker pattern.
- Altered osteocyte lacunar morphology characterizes aseptic loosening.
- Synovial sclerostin shows diagnostic potential with an area under the curve of 0.82.

## Strengths and limitations

- This is the first study presenting an analysis of synovial fluid proteomics with bone histomorphometry, quantitative backscattered electron imaging, and immunofluorescence in aseptic loosening.
- This is an exploratory, single-centre study with a modest sample size.

## Introduction

Total joint replacement is among the most successful procedures in orthopaedic surgery, reliably providing pain relief and restoring function.^1,2^ The demand for primary hip and knee arthroplasty is projected to rise over the coming decades, and with higher procedure volumes, the absolute number of revision surgeries will inevitably also rise.^3-5^ One of the leading indications for revision surgery after hip and knee arthroplasty is aseptic loosening.^6,7^ Aseptic loosening is defined as implant loosening in the absence of periprosthetic joint infection (PJI), and is attributed to inflammation from wear debris that promotes macrophage activation, osteoclastogenesis, and periprosthetic osteolysis.^8-11^

However, despite its prevalence and socioeconomic impact, diagnosis and management remain challenging, and are primarily based on patient history as well as clinical and radiological findings, which are often non-specific and can overlap with other causes of implant failure, such as instability, malalignment, or mechanical wear.^7,12^ Although the involvement of chronic inflammation and dysregulated bone remodelling are recognized as factors in aseptic loosening, the precise molecular and cellular mechanisms remain incompletely understood.^13-15^ In particular, the local biochemical environment of the joint, reflected in the synovial fluid, has not been thoroughly characterized in this condition, nor has it been compared with patients with primary osteoarthritis (OA) or other indications for aseptic revision surgery (e.g. instability).

This knowledge gap may limit the establishment of diagnostic biomarkers and targeted preventive strategies. Biomarkers that are capable of distinguishing aseptic loosening from other aseptic causes of revision surgery could substantially improve early diagnosis, further guide the development of targeted preventive or therapeutic approaches, and help improve the understanding of the general pathophysiology of aseptic loosening. To address this, we performed a detailed analysis of synovial fluid from patients undergoing revision arthroplasty for aseptic loosening and compared it with synovial fluid from patients with primary OA and from those with other aseptic revision indications. We aimed to provide insights into the pathophysiology of aseptic loosening, and to explore candidate diagnostic parameters in the synovial fluid.

## Methods

### Study cohort

In this single-centre prospective exploratory study, we included three groups: 1) patients with primary OA undergoing primary total hip arthroplasty (THA) or total knee arthroplasty (TKA) (n = 13); 2) patients who had radiologically and clinically confirmed aseptic loosening undergoing revision arthroplasty (n = 15); and 3) patients undergoing aseptic revision surgery for other indications (n = 12). The diagnosis of aseptic loosening was established by consensus of the treating arthroplasty surgeons based on the combination of: 1) clinical symptoms consistent with implant loosening; 2) radiological signs of loosening (e.g. progressive radiolucent lines, component migration/subsidence, osteolysis, or change in implant position); and a mandatory 3) intraoperative confirmation of loss of fixation at the implant-bone interface. In both revision groups, infection was excluded by joint aspiration with synovial cytology, as well as by a subsequent microbiological and histological diagnostic work-up according to the criteria of the international consensus meeting 2018.^16^ Patients undergoing aseptic revision surgery for indications other than loosening had no radiological or intraoperative evidence of loosening.

In the aseptic revision group (n = 12), the leading indication was instability (n = 6; 50%), followed by disease progression, i.e., conversions to total joint replacement (n = 2; 16.7%), and single cases of periprosthetic fracture, arthrofibrosis, psoas impingement, and maltracking. The study was approved by the local ethics committee (Ärztekammer Hamburg; 2024-101251-BO-ff).

### Routine laboratory evaluation

Routine laboratory assessments were performed preoperatively in all patients included in the study, including serum measurements of CRP and whole blood white blood cell (WBC) count, as previously described.^17^ Furthermore, synovial fluid was collected via joint aspiration preoperatively from patients who were scheduled for revision surgery, to exclude PJI. For cytological analysis, the aspirate was transferred into ethylenediaminetetraacetic acid (EDTA) tubes and treated with hyaluronidase to reduce viscosity. Total synovial WBC counts and differential leucocyte distributions were subsequently analyzed in the institutional laboratory.

### Synovial fluid biomarker analysis

For synovial biomarker analysis, synovial fluid was aspirated intraoperatively under sterile conditions. The collected samples were immediately centrifuged, and the supernatant was aliquoted and stored at −80°C until analysis. Biomarker concentrations were quantified using the MILLIPLEX MAP Human Bone Magnetic Bead Panel (HBNMAG-51K-13, Merck, Germany) on a Luminex 200 system (Luminex, USA). The assay was performed according to the manufacturer’s protocol with minor modifications for synovial fluid, including an additional standard point and a 1:5 dilution. The 13-plex panel included: interleukin 1-β (IL-1β), IL-6, tumour necrosis factor-α (TNF-α), osteocalcin, osteopontin, osteoprotegerin, dickkopf-related protein 1 (DKK1), sclerostin, fibroblast growth factor-23 (FGF-23), adrenocorticotropic hormone (ACTH), parathyroid hormone (PTH), insulin, and leptin.

### Human tissue biopsies

Trabecular bone biopsies for tissue-based analyses were obtained intraoperatively. During primary arthroplasty, trabecular bone biopsies were collected from the endosteal femoral bone surface in a standardized metaphyseal trabecular region of the femoral neck and distal femoral metaphysis for THA and TKA, respectively. During aseptic revision procedures, representative metaphyseal trabecular bone fragments were harvested directly from the implant-bone interface during prosthesis explantation and routine interface debridement using a standardized approach. The gross size of the harvested bone specimens varied according to the surgical situation and the amount of safely retrievable trabecular/interface bone. No additional bone removal beyond the requirements of the surgical procedure was performed. Therefore, only cases with sufficient sample quality and a size of at least 5 × 5 mm were included in the tissue-based analyses, resulting in a smaller sample size of primary OA (n = 11), aseptic loosening (n = 7), and aseptic revision (n = 7). After fixation in 4% phosphate-buffered formaldehyde, all samples were processed using a diamond-coated saw (EXAKT Advanced Technologies, Germany) to obtain representative trabecular bone specimens for further analyses. One subspecimen was processed for undecalcified histology and embedded in polymethylmethacrylate (PMMA), whereas the second was decalcified in an EDTA-based solution (Usedecalc; Medite, USA) and embedded in paraffin.

### Histological and osteocyte lacunar analysis

For histological analysis, 4 µm-thick sections were prepared and stained with toluidine blue and von Kossa, as previously described.^18^ Histomorphometry of the trabecular bone compartment was performed using an OsteoMeasure System (OsteoMetrics, USA) in accordance with the guidelines of the American Society for Bone and Mineral Research.^19^ The number and size of osteocyte lacunae were evaluated using quantitative backscattered electron imaging (QBEI) with a scanning electron microscope (LEO 435; LEO Microscopy, UK) equipped with a specialized backscattered electron detector (Type 202; K.E. Developments, UK), as previously described.^20^ For each specimen, regions of interest covering on average 1 mm^2^ of trabecular bone area were evaluated. Images were acquired at an accelerating voltage of 20 kV and a beam current of 680 pA at a constant working distance of 20 mm and a magnification of 120×. Signal amplification (brightness and contrast) was calibrated throughout the procedure using carbon and aluminium standards. Osteocyte lacunar analysis was performed on calibrated greyscale images using a custom MATLAB script (MathWorks, USA). Lacunae were identified using a predefined grey-level threshold and size filter (8 to 90 µm^2^). Objects smaller or larger than this range were discarded. Following automated segmentation, the identified objects were manually reviewed to verify plausibility and exclude misclassifications, artefacts, and non-lacunar structures.

### Sclerostin immunofluorescence

Immunofluorescence (IF) was performed on decalcified 4 µm-thick sections (n = 4 per group), which were deparaffinized, rehydrated, and subjected to gentle heat-induced epitope retrieval in 10 mM citrate buffer (pH 6.0; 85°C, 15 mins). Sections were blocked for one hour at room temperature (RT) in 5% normal donkey serum (NDS). Staining was performed overnight at 4°C with rabbit anti-sclerostin (21933-1-AP, 1:100; Proteintech, USA) and goat anti-CD31 (#AF3628, 1:100; R&D Systems, UK). This was followed by donkey anti-goat Alexa Fluor 647 (#A32849, 1:400, one hour at RT; Thermo Fisher Scientific, USA) and donkey anti-rabbit Alexa Fluor 488 (#A32790, 1:400, one hour at RT; Thermo Fisher Scientific). Slides were mounted using Fluoroshield mounting medium (#F6182; Sigma-Aldrich, USA). Images were acquired using an Olympus FV3000 (Japan) confocal microscope.

### Statistical analysis

All statistical analyses were conducted using R and GraphPad Prism v. 10.4 (GraphPad Software, USA). The Shapiro-Wilk test was applied to assess the normality of continuous variables. For comparisons between two groups, the independent-samples *t*-test was used for normally distributed data, and the Mann-Whitney U test for non-normally distributed data. For comparisons across more than two groups, analysis of variance (ANOVA) followed by Tukey’s post hoc test was used for normally distributed data, while the Kruskal-Wallis test followed by Dunn’s post hoc test was used for non-parametric data. Differences in categorical variables were assessed using either Fisher’s exact or chi-squared test. Furthermore, for diagnostic performance analysis, receiver operating characteristic (ROC) curves and the area under the curve (AUC) were calculated for synovial biomarkers within the revision synovial biomarker subset to assess discrimination of aseptic loosening from other aseptic revision indications. For synovial sclerostin, an exploratory cut-off was selected using the maximal Youden index, and sensitivity, specificity, positive predictive value (PPV), and negative predictive value (NPV) were calculated at that threshold. A significance level of 0.05 was used.

## Results

### Routine blood and synovial parameters cannot identify aseptic loosening

A total of 40 patients were included across the three different groups: primary OA, aseptic loosening, and other aseptic revision (Figure 1a). There were no differences regarding demographic variables, including age, sex, and BMI (Table I, Figures 1b and 1c). Additionally, there was a balanced distribution between knee and hip joints across the study cohort (Table I). To gain further insights into potential factors associated with aseptic loosening, routine systemic inflammatory markers were first assessed. Here, whole blood WBC count and serum CRP levels showed no differences between groups (Figures 1d and 1e). Both parameters were within normal or near-normal ranges in the majority of patients. Thus, routine systemic markers could not differentiate aseptic loosening from primary OA or other aseptic revisions.

**Fig. 1:**
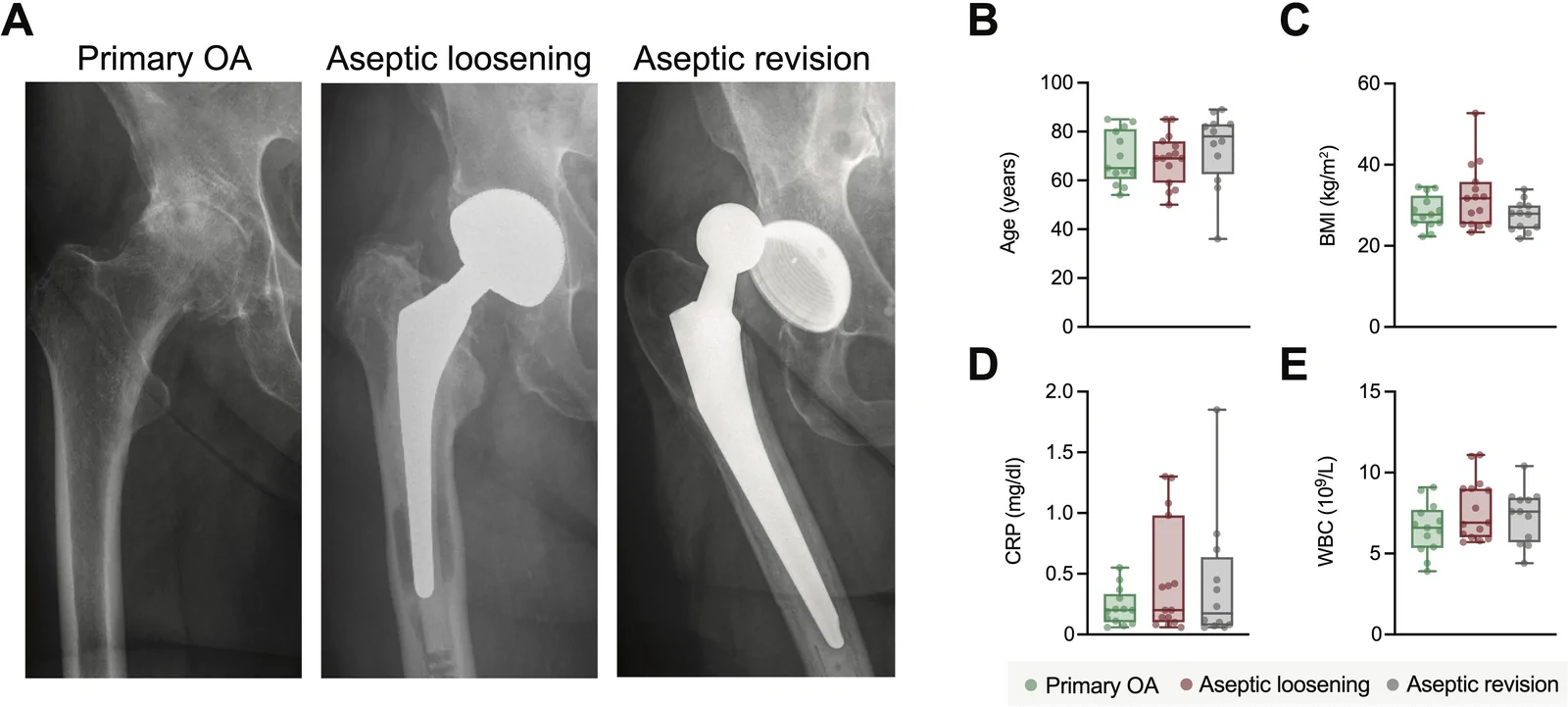
Demographic and inflammatory markers do not differentiate aseptic loosening. a) Representative anteroposterior hip radiographs from patients with primary osteoarthritis (OA), aseptic loosening, and aseptic revision (e.g. recurrent dislocation). Quantification of b) age, c) BMI, d) serum CRP, and e) peripheral white blood cell (WBC) count across the three groups. Statistical analysis was performed using one-way analysis of variance with Tukey’s post hoc test or the Kruskal-Wallis test with Dunn’s multiple comparisons, as appropriate.

**Table I.** Demographic characteristics of the patient cohort.

Characteristic	Overall (n = 40)	Primary OA (n = 13)	Aseptic loosening (n = 15)	Aseptic revision (n = 12)	p*-*value
Mean age, yrs (SD)	70.3 (13.0)	69.3 (10.9)	68.8 (10.4)	73.3 (15.5)	0.609*
Mean BMI, kg/m^2^ (SD)	29.4 (6.0)	28.3 (4.1)	32.0 (7.9)	27.3 (3.7)	0.193†
**Sex, n (%)**					0.199‡
Female	25 (62.5)	9 (69.2)	11 (73.3)	5 (41.7)	
Male	15 (37.5)	4 (30.8)	4 (26.7)	7 (58.3)	
**Affected joint, n (%)**					0.705‡
Hip	22 (55.0)	8 (61.5)	7 (46.7)	7 (58.3)	
Knee	18 (45.0)	5 (38.5)	8 (53.3)	5 (41.7)	

* One-way analysis of variance.

† Kruskal-Wallis test.

‡ Chi-squared test.

OA, osteoarthritis.

As a next step, routine synovial cytology was performed on preoperatively collected joint aspirates from patients included in the aseptic loosening and aseptic revision cohorts. Total synovial WBC counts were low across both groups and did not differ between aseptic loosening and other aseptic revisions (Figure 2a). Differential analysis revealed similar proportions of polymorphonuclear neutrophils (PMN), lymphocytes, and monocytes in both revision groups (Figures 2b to 2d). Hence, synovial cytology provides no additional diagnostic value in distinguishing between forms of aseptic revision.

**Fig. 2:**
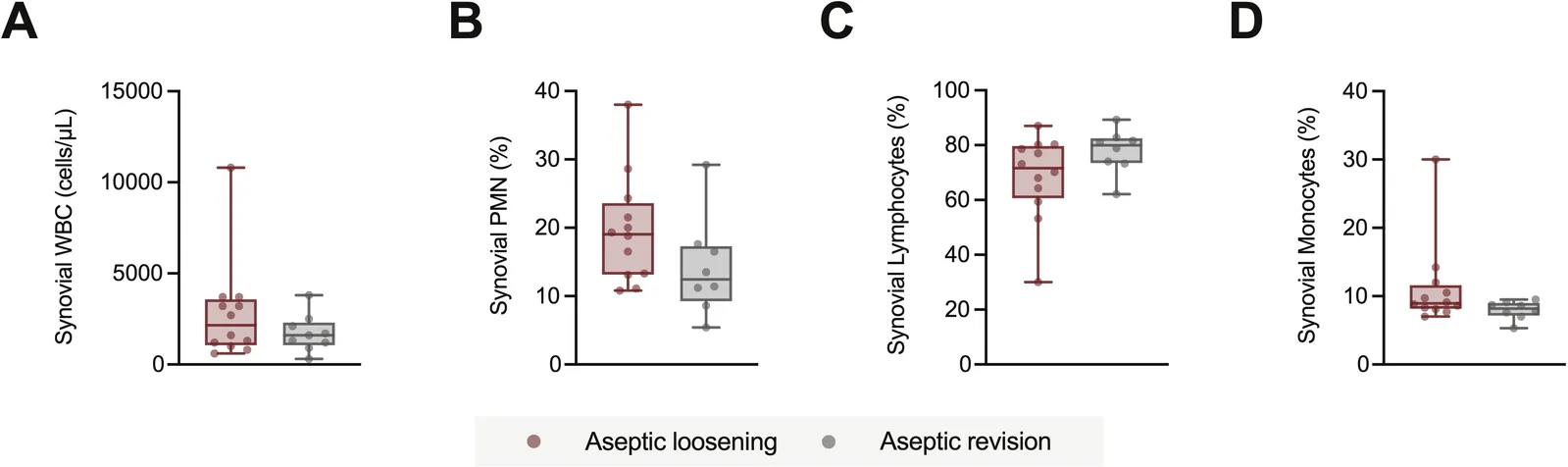
Routine synovial cytology does not distinguish aseptic loosening from other indications for aseptic revision surgery. a) Quantification of synovial white blood cell (WBC) count in the two groups. Synovial differential proportions of b) polymorphonuclear neutrophils (PMN), c) lymphocytes, and d) monocytes in patients with aseptic loosening versus other aseptic revision indications. Statistical analysis was conducted using the independent-samples *t*-test or the Mann-Whitney U test, as appropriate.

### Synovial sclerostin is decreased in aseptic loosening

As there were no group differences evident in routine synovial analysis, we conducted a multiplex protein analysis to identify disease-specific biochemical signatures in the synovial environment (Figure 3a). The panel included key regulators of bone turnover (osteoprotegerin, sclerostin, DKK1, osteocalcin, osteopontin), inflammatory cytokines (IL-1β, IL-6, TNF-α), and metabolic proteins like leptin. Compared with primary OA, synovial fluid from patients with aseptic loosening showed lower levels of osteocyte-derived inhibitors of bone formation. Specifically, concentrations of DKK1 and sclerostin were reduced (Figure 3b). When comparing aseptic loosening with other indications for aseptic revision surgery, most synovial parameters were comparable with the notable exception of DKK1 and sclerostin (Figure 3c). Both aseptic loosening and other indications for aseptic revision showed a marked reduction in osteoprotegerin levels compared with primary OA (aseptic loosening vs primary OA: p < 0.001; Kruskal-Wallis test with Dunn’s post hoc test; aseptic revision vs primary OA: p = 0.001; Kruskal-Wallis test with Dunn’s post hoc test) (Figure 3d). In addition, osteopontin levels were increased in both aseptic groups relative to primary OA (aseptic loosening vs primary OA: p < 0.001; Kruskal-Wallis test with Dunn’s post hoc test; aseptic revision vs primary OA: p = 0.017; Kruskal-Wallis test with Dunn’s post hoc test) (Figure 3e). Most notably, the analysis of the osteocyte-derived proteins DKK1 and sclerostin revealed unique patterns in aseptic loosening. Among the synovial biomarkers, sclerostin showed the clearest group separation. Synovial sclerostin levels were significantly lower in aseptic loosening than in OA (p = 0.026; Kruskal-Wallis test with Dunn’s post hoc test) and other aseptic revisions (p = 0.029; Kruskal-Wallis test with Dunn’s post hoc test), whereas OA and other aseptic revisions did not differ. DKK1 showed a similar directional pattern, with lower levels in aseptic loosening than in OA (p = 0.001; Kruskal-Wallis test with Dunn’s post hoc test), but the difference between aseptic loosening and other aseptic revisions did not reach statistical significance after multiple-comparison correction (p = 0.057; Kruskal-Wallis test with Dunn’s post hoc test) (Figures 3f and 3g). Together, these findings identify synovial sclerostin as the strongest osteocyte-related biomarker associated with aseptic loosening, while DKK1 showed a concordant but weaker reduction.

**Fig. 3:**
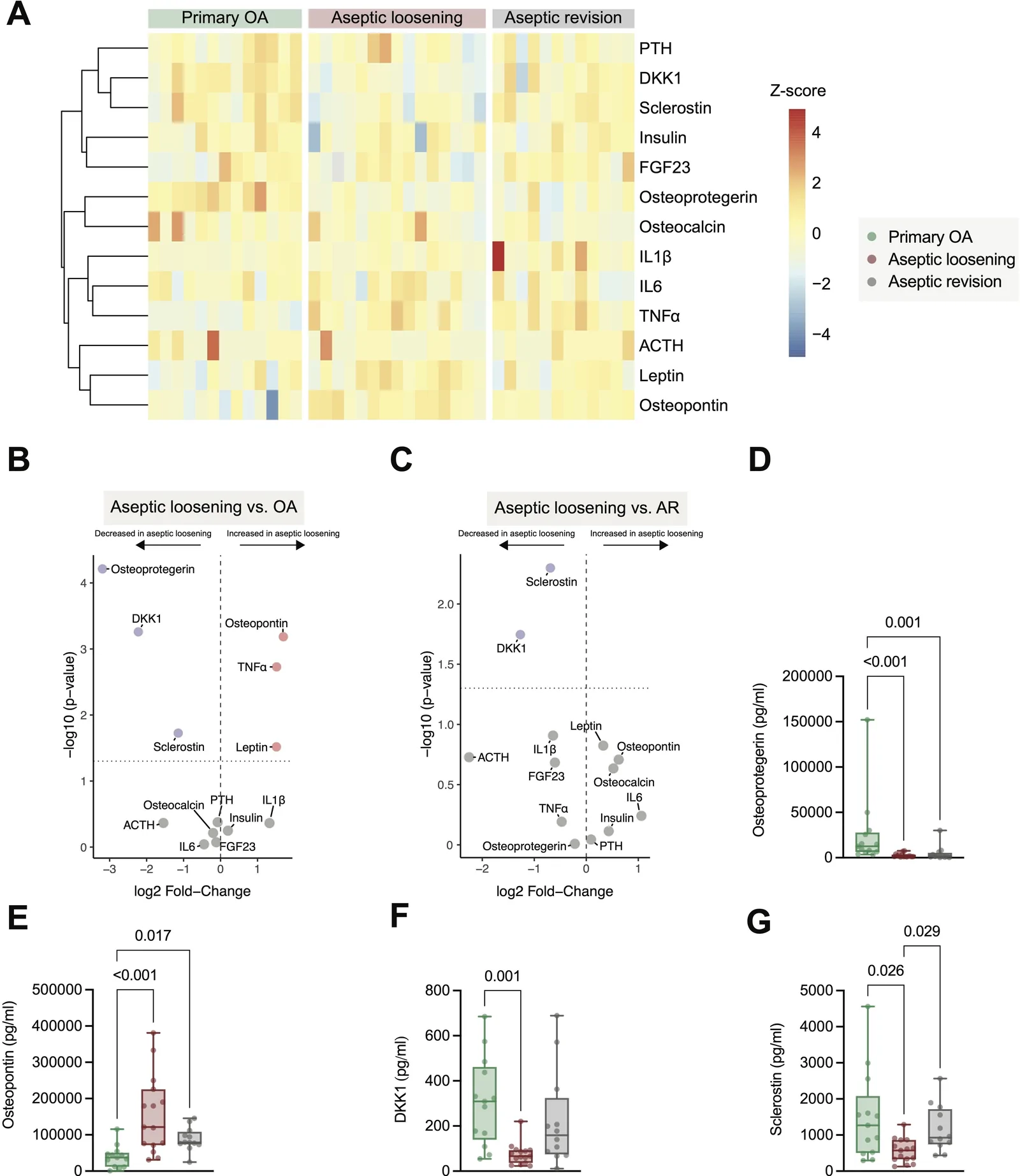
Multiplex synovial biomarker analysis reveals a unique protein signature characterized by decreased sclerostin levels in aseptic loosening. a) Heatmap displaying the expression levels of 13 proteins in the synovial fluid of the three groups, as standardized row z-scores. b) Visualization of differentially expressed proteins in the synovial fluid of patients with aseptic loosening compared with primary osteoarthritis (OA). c) Visualization of differentially expressed proteins in the synovial fluid of patients with aseptic loosening compared with other indications for aseptic revision (AR) surgery. Quantification of d) osteoprotegerin, e) osteopontin, f) dickkopf-related protein 1 (DKK1), and g) sclerostin across the three groups. Statistical analysis was performed using one-way analysis of variance with Tukey’s post hoc test or the Kruskal-Wallis test with Dunn’s multiple comparisons, as appropriate. Exact p-values are reported for significant comparisons unless p < 0.001. ACTH, adrenocorticotropic hormone; FGF23, fibroblast growth factor-23; IL-1β, interleukin-1β; PTH, parathyroid hormone; TNFα, tumour necrosis factor α.

### Histomorphometry reveals preserved microstructure in aseptic loosening

Next, we asked whether the detected alterations in local synovial metabolism translate into tissue-level skeletal changes in aseptic loosening. We therefore performed bone histomorphometry on trabecular bone specimens (Figure 4a). Here, no differences were evident in bone mass and trabecular microarchitecture compared with primary OA and other aseptic revisions (Figure 4b, Supplementary Figure 1a-1c). Furthermore, no differences were evident in the proportion of bone surface covered by osteoid or in osteoid thickness (Figure 4c, Supplementary Figure 1d). Cellular analysis also revealed no differences in the number of osteoblasts and osteoclasts (Figures 4d and 4e). Thus, despite clear differences in synovial bone metabolism markers, quantitative bone histomorphometry showed no between-group differences in trabecular microstructure or turnover.

**Fig. 4:**
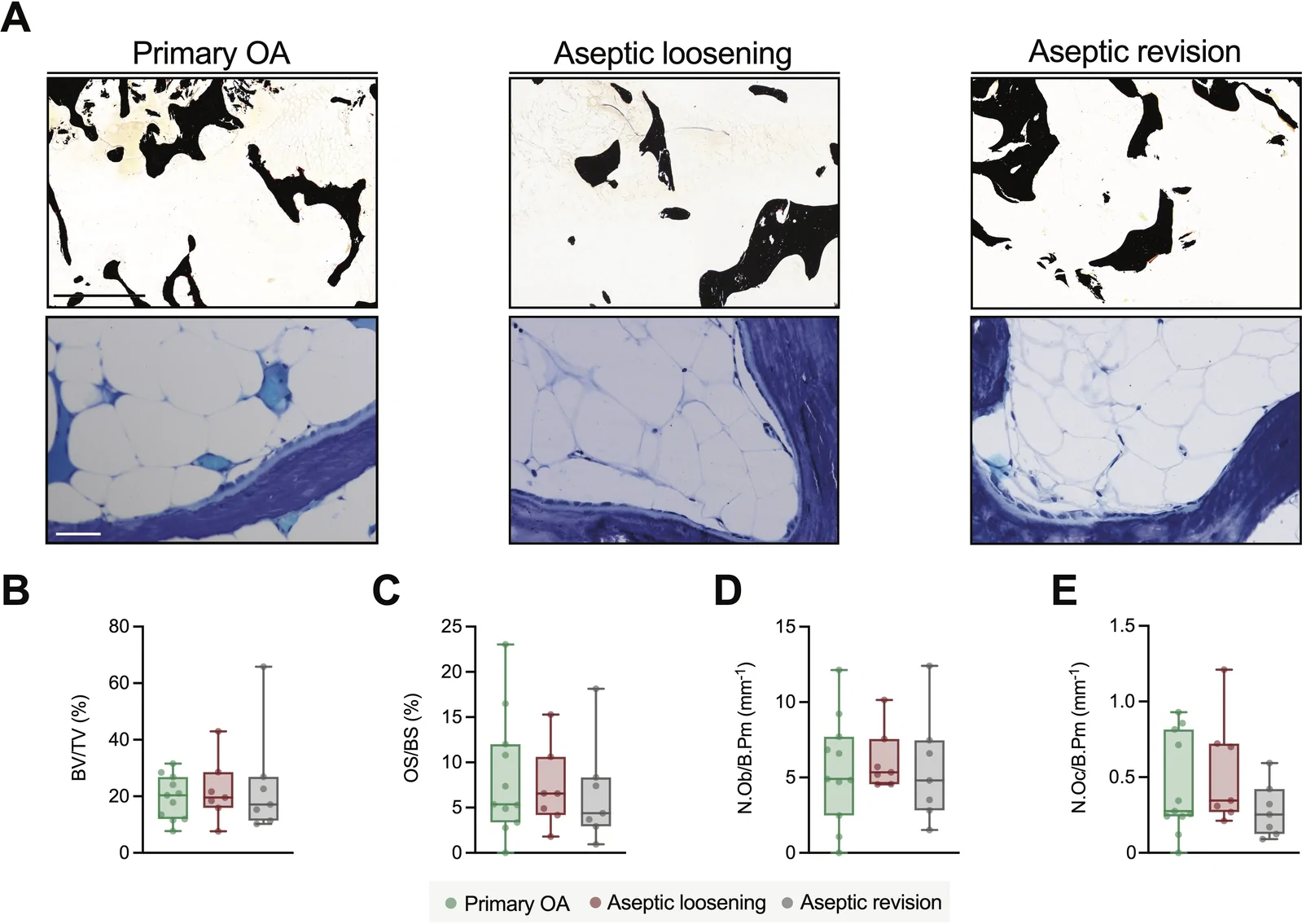
Trabecular bone histomorphometry indicates no difference in bone structure and remodelling. a) Representative images of von Kossa (upper panel) and toluidine blue-stained (lower panel) sections of patients with primary osteoarthritis (OA), aseptic loosening, and other aseptic revision indications. Scale bars: 1 mm (upper panel); 50 µm (lower panel). Histomorphometric quantification of b) bone volume fraction (BV/TV), c) osteoid surface per bone surface (OS/BS), d) number of osteoblasts, and e) osteoclasts per bone perimeter across the three groups. Statistical analysis was performed using one-way analysis of variance with Tukey’s post hoc test or the Kruskal-Wallis test with Dunn’s multiple comparisons, as appropriate.

### Enlarged osteocyte lacunae and reduced sclerostin abundance signify aseptic loosening

Given that osteocyte-derived proteins were altered in the synovial fluid of patients with aseptic loosening, we next analyzed osteocyte properties (Figure 5a). QBEI-based analysis showed a comparable osteocyte lacunar density across groups, whereas the osteocyte lacunar area was specifically increased in patients with aseptic loosening (p = 0.043; Kruskal-Wallis test with Dunn’s post hoc test) (Figure 5b, Supplementary Figure 2). To link these findings to osteocyte function on a tissue level, we performed IF in the trabecular bone compartment and confirmed reduced sclerostin expression in aseptic loosening compared with primary OA (p = 0.043; Kruskal-Wallis test with Dunn’s post hoc test) (Figures 5c and 5d). Thus, the synovial fluid signature of low sclerostin is mirrored by decreased sclerostin abundance in bone.

**Fig. 5:**
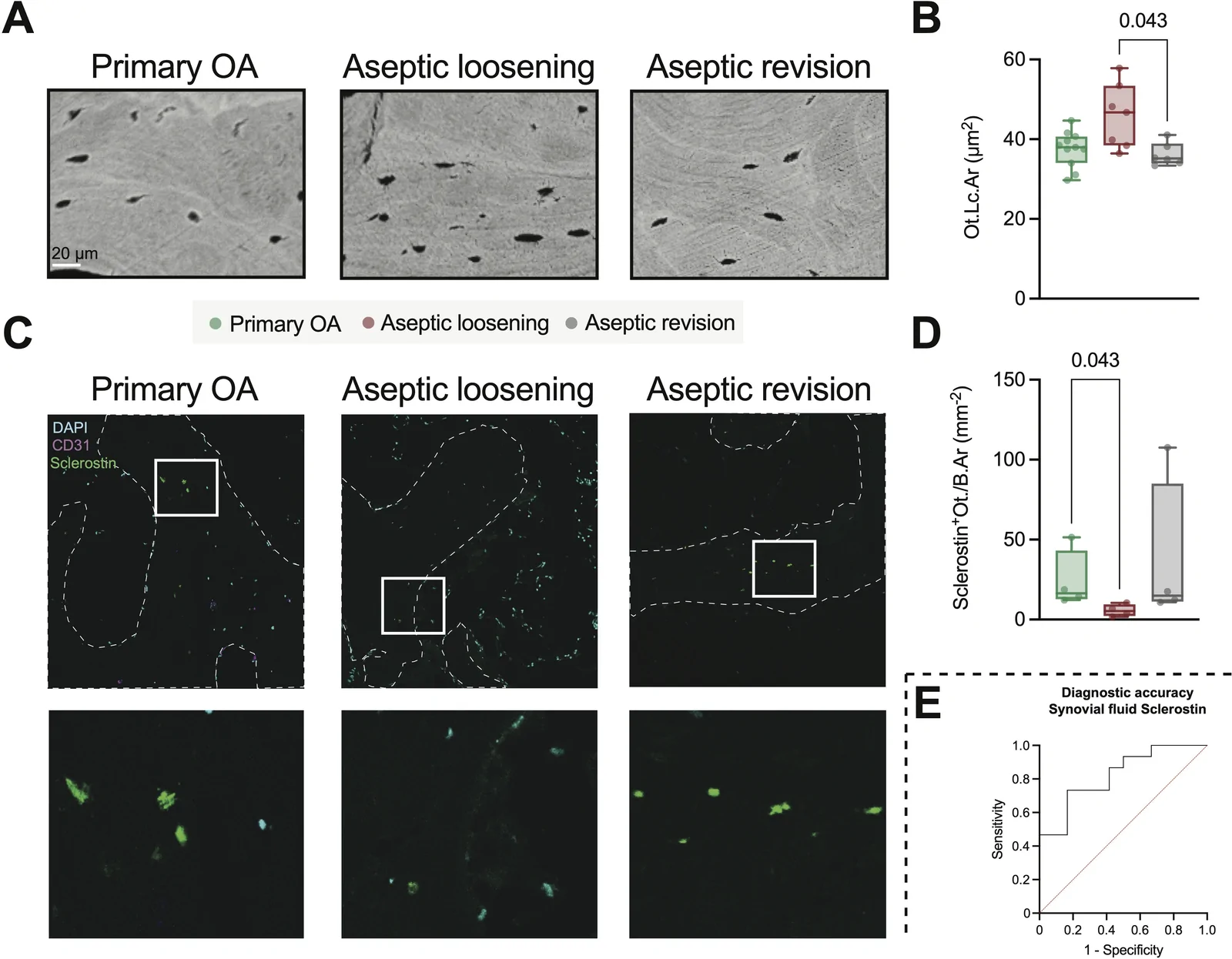
Osteocytes in patients with aseptic loosening are characterized by morphological alterations and decreased sclerostin expression. a) Representative images derived from quantitative backscattered electron imaging of patients with primary osteoarthritis (OA), aseptic loosening, and other indications for aseptic revision surgery. Scale bar: 20 µm. b) Quantification of the osteocyte lacunar area (Ot.Lc.Ar) across the three groups. c) Representative images of sections stained for CD31 and sclerostin through immunofluorescence across the three groups. Dashed lines indicate boundaries of the trabecular bone. d) Quantification of sclerostin-positive osteocytes per bone area (Sclerostin^+^Ot./B.Ar). e) Visualization of the diagnostic accuracy of local sclerostin in the synovial fluid using the area under the receiver operating characteristic curve. Statistical analysis was performed using one-way analysis of variance with Tukey’s post hoc test or the Kruskal-Wallis test with Dunn’s multiple comparisons, as appropriate. Exact p-values are reported for significant comparisons unless p < 0.001.

From a clinical standpoint, we further evaluated the diagnostic performance of synovial sclerostin for identifying aseptic loosening. Within the revision synovial biomarker subset, synovial sclerostin discriminated aseptic loosening from other aseptic revision with an AUC of 0.82 (95% CI 0.67 to 0.98; p = 0.005). Using the exploratory Youden-optimal cut-off of < 705.9 pg/ml, sensitivity was 73.3% (95% CI 48.1 to 89.1), specificity 83.3% (95% CI 55.2 to 97.0), PPV 84.6%, and NPV 71.4%.

Thus, synovial sclerostin may serve as a biomarker for differentiating aseptic loosening from other revision indications (Figure 5e).

## Discussion

Aseptic loosening represents a leading cause of revision after arthroplasty.^6,21^ Despite its high incidence and substantial patient burden, pathophysiological insights, as well as derived preventive, diagnostic, and therapeutic strategies, remain limited. In this study, we provide evidence supporting a potential role for osteocytes in the pathophysiology of aseptic loosening. More specifically, we identified that sclerostin, an osteocyte-derived inhibitor of Wnt signalling, was reduced in both the synovial fluid and the periprosthetic bone tissue. Together, our data suggest that osteocytes adapt to stress at the implant-bone interface in response to local mechanical and inflammatory changes. Sclerostin downregulation may function as a compensatory mechanism that counteracts the osteolytic stimulus in aseptic loosening and supports peri-implant bone formation.

We first confirmed that routine blood inflammatory markers, including WBC and CRP, are unable to distinguish patients with aseptic loosening from those with primary OA or from those with other indications for aseptic revision surgery. This aligns with prior observations that CRP levels in aseptic loosening are typically normal or only marginally elevated.^22^ Furthermore, routine synovial analysis failed to identify any distinct alterations in the synovial fluid. Namely, synovial WBC count and differential were unable to differentiate aseptic loosening from other aseptic revisions, indicating that it provides no diagnostic benefit in this case. This is consistent with earlier studies demonstrating no differences in synovial WBC counts between controls and patients with aseptic loosening, as well as no variation in the indications for aseptic revision.^23,24^ Notably, some studies have reported higher synovial WBC counts in polyethylene wear than in aseptic loosening.^25,26^ However, our cohort did not include patients with isolated polyethylene wear, which explains why synovial WBC and differential did not differentiate aseptic loosening from other aseptic revisions. Therefore, our findings reinforce that standard serum and synovial markers lack diagnostic utility for identifying aseptic loosening or differentiating it from other aseptic revision aetiologies.

Most importantly, our findings implicate osteocytes as central contributors to the process of aseptic loosening. We observed lower expression of the osteocyte-derived protein sclerostin in both the synovial fluid and periprosthetic bone tissue. In addition, patients with aseptic loosening exhibited larger osteocyte lacunae, with no differences in lacunar density. This morphological pattern is consistent with perilacunar remodelling (PLR).^27,28^ At first glance, low sclerostin levels alongside morphological features consistent with PLR seem counterintuitive, as sclerostin can upregulate proteins associated with PLR.^29,30^ However, sclerostin is strongly load-sensitive and can be downregulated by increased mechanical loading.^31^ We can only speculate that progressive micromotion and abnormal strain at a loose implant-bone interface would suppress sclerostin and DKK1 expression, thereby enabling local Wnt signalling to increase bone formation. Furthermore, osteocytes exposed to ultra-high-molecular-weight polyethylene particles upregulate PLR-associated enzymes (e.g., tartrate-resistant acid phosphatase, cathepsin K), reinforcing that periprosthetic osteolysis is not solely osteoclast-driven.^32^ Notably, DKK1 showed a concordant but weaker reduction, reaching significance versus primary OA but not clearly separating aseptic loosening from aseptic revision after multiple-comparison correction. This pattern is consistent with broader osteocyte-related modulation of extracellular Wnt inhibition in aseptic loosening. Consistent with this, a rat model of periprosthetic osteolysis and aseptic loosening showed downregulation of *Sost* and *Dkk1* in periprosthetic bone.^33^ The reduced synovial and tissue sclerostin observed in aseptic loosening may be interpreted, in the context of our study, as a local response at the periprosthetic bone interface rather than a simple consequence of reduced global mobility. Periprosthetic microinstability, altered local strain, osteocyte distress, and inflammatory signalling may jointly modulate Wnt antagonism in this setting. However, related processes may also be present in other failure modes, including infection-associated loosening; therefore, the present study does not establish specificity for aseptic loosening. Septic loosening/PJI was not included in the present cohort, although infection-associated periprosthetic bone pathology has also been linked to osteocyte involvement, including intracellular *Staphylococcus aureus* persistence.^34^ Broader PJI studies further support a persistent inflammatory and altered bone-remodelling phenotype that may contribute to later loosening after infection.^35,36^ At the same time, osteoprotegerin was lower, implying increased receptor activator of nuclear factor κB ligand (RANKL)-driven resorptive stimuli. Together, these findings point to dysregulated coupling of formation and resorption at the implant-bone interface. Clinically, reduced synovial sclerostin could serve as an adjunctive biomarker for aseptic loosening, particularly useful given that current diagnostics still rely heavily on clinical and radiological findings, which are often inconclusive. Additionally, osteocyte-targeted therapies (e.g., strategies that modulate Wnt signalling or osteocyte activity) could be examined in the future as potential preventive approaches. The most immediate potential clinical value of synovial sclerostin is not as a standalone diagnostic test or a substitute for standard imaging and infection work-up, but rather as an adjunctive biomarker in painful or diagnostically unresolved arthroplasty failure after infection has been excluded. In this setting, a low synovial sclerostin signal, with a concordant but weaker DKK1 pattern, may help biologically characterize aseptic loosening and distinguish it from other aseptic revision indications. If validated in larger cohorts and in preoperative aspirates, such biomarkers could improve patient stratification and reduce diagnostic uncertainty. Beyond diagnosis, these findings raise the possibility that osteocyte-/Wnt-directed pathways may be relevant therapeutic targets in aseptic loosening. Notably, a previous study reported in a rat model of particle-induced aseptic loosening that systemic anti-sclerostin antibody prevents implant loosening by stimulating bone formation and inhibiting bone resorption.^37^ However, the present study is exploratory and cross-sectional, and reduced sclerostin in aseptic loosening does not establish causality or imply immediate clinical benefit from anti-sclerostin treatment. Any therapeutic extrapolation, therefore, remains hypothesis-generating only, and requires mechanistic studies, validation in preoperative synovial aspirates, and prospective clinical investigation.

Our study has several limitations. First, it is a small, single-centre exploratory cohort. However, this is the first study to perform synovial phenotyping as well as tissue analyses in patients with aseptic loosening, thereby providing novel insights. Second, the cross-sectional design prevents causal inference or temporal sequencing of biomarker changes. Thus, longitudinal sampling will be needed to determine trajectories and their impact on patients. Third, we did not perform systemic biomarker profiling. While synovial fluid likely reflects the periprosthetic milieu, paired serum/plasma profiling is needed to validate generalizability.

A further important limitation is that septic loosening/PJI was not included as a comparator group. Thus, while infection was excluded according to the institutional preoperative and intraoperative work-up, we cannot determine whether the observed low synovial sclerostin profile, together with the weaker DKK1 pattern, is specific to aseptic loosening or may also occur in infection-associated implant loosening. This is clinically relevant because, once loosening is suspected radiologically, the key diagnostic question is often not whether loosening exists, but whether it is septic or aseptic. Accordingly, our findings should not be interpreted as evidence that these biomarkers can replace standard infection diagnostics. Future prospective studies should therefore directly compare aseptic loosening with septic loosening/PJI, using standardized preoperative and intraoperative infection criteria and matched aspiration-based sampling, to define the specificity and real clinical utility of these biomarkers.

In conclusion, our data support a central role of osteocytes in the pathophysiology of aseptic loosening and indicate that low sclerostin levels may represent a novel biomarker for this condition. Larger, prospective studies that integrate synovial and systemic biomarkers with periprosthetic tissue phenotyping are needed to clarify mechanisms and validate clinical utility.

## Data Availability

The data that support the findings of this study are available from the corresponding author upon reasonable request.
